# Discovery of a Novel Periodontal Disease-Associated Bacterium

**DOI:** 10.1007/s00248-018-1200-6

**Published:** 2018-06-02

**Authors:** Pedro J. Torres, John Thompson, Jeffrey S. McLean, Scott T. Kelley, Anna Edlund

**Affiliations:** 1 Department of Biology, San Diego State University, San Diego, CA 92182 USA; 2 Department of Periodontics, University of Washington School of Dentistry, Seattle, WA 98195 USA; 3 J. Craig Venter Institute, Genomic Medicine Group, La Jolla, CA 92037 USA

**Keywords:** Periodontitis, Metagenomics, Oral microbiome, Bacteroidetes, Candidate phyla

## Abstract

One of the world’s most common infectious disease, periodontitis (PD), derives from largely uncharacterized communities of oral bacteria growing as biofilms (a.k.a. plaque) on teeth and gum surfaces in periodontal pockets. Bacteria associated with periodontal disease trigger inflammatory responses in immune cells, which in later stages of the disease cause loss of both soft and hard tissue structures supporting teeth. Thus far, only a handful of bacteria have been characterized as infectious agents of PD. Although deep sequencing technologies, such as whole community shotgun sequencing have the potential to capture a detailed picture of highly complex bacterial communities in any given environment, we still lack major reference genomes for the oral microbiome associated with PD and other diseases. In recent work, by using a combination of supervised machine learning and genome assembly, we identified a genome from a novel member of the Bacteroidetes phylum in periodontal samples. Here, by applying a comparative metagenomics read-classification approach, including 272 metagenomes from various human body sites, and our previously assembled draft genome of the uncultivated *Candidatus Bacteroides periocalifornicus* (CBP) bacterium, we show CBP’s ubiquitous distribution in dental plaque, as well as its strong association with the well-known pathogenic “red complex” that resides in deep periodontal pockets.

**Electronic supplementary material:**

The online version of this article (10.1007/s00248-018-1200-6) contains supplementary material, which is available to authorized users.

## Introduction

Initial studies of periodontitis (PD) relied on culturing methods and traditional culture independent methods (i.e., DNA-DNA hybridization, cloning, and targeted sequencing) [1, 2], neither of which allow microbial diversity to be fully understood. The advent of culture independent high-throughput sequencing technology has increased our understanding of the diversity of oral bacteria through two commonly used approaches: sequencing of conserved 16S ribosomal RNA genes and untargeted (“shotgun”) sequencing of all (“meta”) microbial genomes (“genomics”). However, because we lack reference genome sequence data for large portions of the microbial tree of life, there remains a high potential for overlooking microbes that are truly present in any given environment. To fill in some of these knowledge gaps and bypass the need for sequence homology for taxonomic classification, studies have employed contig binning, i.e., short reads assembled into contiguous sets of overlapping reads (contigs), which can be grouped into taxa based on sequence composition, similarity or read coverage [3]. One such approach includes supervised binning, which assigns contigs into taxonomic classes using a model trained with available reference sequences [4]. In a previous study, we employed this methodology on metagenomics sequence data obtained from microbial samples collected from 12 subjects with severe periodontal disease [5]. Briefly, all quality-trimmed reads were de novo assembled using SPAdes v 2.40 [6, 7]. SPAdes was chosen as assembly algorithm, since this program has demonstrated exceptionally high genome assembly quality as compared to other available assemblers, both single-cell assemblers as well as assemblers for multi-cell data (e.g. Velvet and SoapDeNovo) [6, 7]. We conducted post-assembly processing of contigs, which included taxonomic classification based on a machine learning algorithm using the MG Taxa tool as described earlier [4]. Several large contigs that were presented in a number of libraries had the same k-mer frequency and were originally classified at a low score to uncultivated phylum OD1, indicating they were distantly related to any previously sequenced genome [4]. These contigs, from a single sampling subject, were then sorted into a bin and further inspected for k-mer frequency consistency and used for downstream genome analyses. The assembled draft genome is 2.53 Mb and consists of 49 major contigs (sizes range between 18,374 to 129,525 bp), with an overall GC content of 59.4% (GenBank accession number: LIIK00000000). Gene annotation using the Prokaryotic Genome Automatic Annotation Pipeline (PGAAP) provided by the National Center for Biotechnology Information (NCBI) identified a total of 1875 genes, consisting of 1678 coding sequences, 39 tRNAs, and 1 rRNA operon (5S) [4]. Due to that metagenomic assembly methodologies cannot distinguish nearly identical sequences, which may originate from different genomes within the sample, our draft genome may represent several closely related bacterial strains.

Uncultivated groups, such as Candidate bacterial phyla are prevalent in the oral cavity, including Saccharibacteria/TM7, Gracilibacteria (GN02), SR1, and WPS-2 clades. Recently, strain TM7x, a member of the elusive TM7 Candidate phylum, associated with severe PD and other inflammatory conditions, was isolated from a human saliva sample [8]. Its cultivation facilitated the sequencing of a complete genome and revealed its clearly symbiotic lifestyle as the genome did not contain any amino acid biosynthetic pathways. Aside from this rare example, the ecological and clinical role of uncultivated bacteria and archaea in PD still remains a challenge.

PD, one of the world’s most common infectious diseases, is a progressive polymicrobial infection that if untreated can progress to moderate and severe periodontitis. Overall, the disease refers to the inflammatory process that occurs in the tissues surrounding the teeth in response to the growth of bacterial biofilms, or dental plaque, along the gumline. Eventually, PD results in the breakdown of the periodontal ligament and alveolar bone, and can lead to loss of teeth. PD affects the majority of adults worldwide and may contribute to various systemic diseases, including atherosclerosis (ATH), cardiovascular disease, type 2 diabetes, and rheumatoid arthritis [9]. Despite decades of research, the substantial differences among periodontitis patients in disease incidence, progressivity, and response to treatment are poorly understood.

Subgingival microbiota of periodontally healthy subjects has been shown to differ from that found in subjects with periodontal disease [10]. Studies also show that there is a striking change in the composition of the microbial profiles with greater disease severity. The shift is particularly marked for the known pathogens in the so-called red complex, i.e., *Porphyromonas gingivalis*, *Treponema denticola*, and *Tannerella forsythia* [11], whose numbers increase with pocket depth [12]. Researchers have found little or no relationship to pocket depth for the majority of other microbial species; however, most members of the so-called orange complex, which includes *Fusobacterium nucleatum* among other species, and all species of the red complex strongly associate with deeper periodontal pockets and disease severity [13]. In fact, the abundances of all members of the red complex are highly correlated, and studies have shown that co-infection with multiple members cause more severe PD than individual infections [11, 14]. The establishment of periodontal biofilms on tooth surfaces is initiated by fast growing bacterial community members of the yellow complex, such as *Streptococcus mitis* and *S. oralis*, while bridging species of the orange complex, i.e., *Fusobacterium* and late colonizers of the red complex require longer periods of time to grow [13]. Co-culturing studies have shown that members of the orange complex, particularly *F. nucleatum*, significantly enhance the growth of the more severe periodontal pathogens in the red complex [15]. It is important to note that most studies require cultivation or rely on reference databases of known sequences (16S rRNA gene and whole genomes); any bacteria that do not readily culture or are missing in the databases are ignored or missed.

Here, we were able to further characterize a recently discovered member of the *Bacteriodetes* phylum, CBP, by analyzing a total of 272 previously published metagenomes from various human body sites representing both healthy adults and adults with PD. We found that CBP is orally ubiquitous, existing in both healthy and diseased individuals, but not present in gut or skin samples. CBP also increases with increased pocket depth, co-exists with both *F. nucleatum*, *T. denticola*, and *P. gingivalis.* Its abundance is strongly correlated with members of the red complex, but not healthy commensals, all of which suggest that CBP is a novel candidate member of the symbiotic and pathogenic red complex.

## Methods

### *CandidatusBacteriodesperiocalifornicus* Draft Genome Information

The draft genome LIIK00000000 was accessed via Bioproject Accession PRJNA289925, Biosample Accession SAMN03859889. Other relevant information (e.g. annotations) associated with the LIIK00000000 genome can be found via NCBI Taxon ID 1702214, IMG Submission ID: 77482, GOLD ID in IMG Database Study ID: Gs0118016 Project ID: Gp0126827, GOLD Analysis Project Id: Ga0104344.

### Maximum Likelihood Tree

Seventy-eight genomes representing major lineages from the *Bacteroidetes* phylum were downloaded from NCBI. Thirty-one taxa-specific marker genes, which were previously determined as single copy genes and unique at the nucleotide level [16] were concatenated and analyzed for optimal tree topography under evolutionary criteria by using the Molecular Evolutionary Genetics Analysis (MEGA) software, version 6.0 [17]. Five thousand bootstrap iterations were performed.

### HMP Dataset

The National Institutes of Health (NIH) Human Microbiome Project (HMP) was established by the NIH Common Fund (http://commonfund.nih.gov/hmp/) to provide a public resource to facilitate human microbiome research [18]. Two hundred and fourteen metagenomes were obtained from the HMP whole metagenomics shotgun sequencing website (https://www.hmpdacc.org/HMIWGS/healthy/). This included 18 gut, 14 left retroauricular crease a.k.a. skin, 6 saliva, 16 subgingival, and 160 supragingival datasets (Table S1).

### Human Oral Microbiome Datasets

Published metagenomic libraries, representative of both healthy and diseased subjects, were obtained from the Human Oral Microbiome Database (HOMD) under the submission number 20130522 (ftp://ftp.homd.org/publication_data/20130522/) and from a study by Duran-Pinedo and colleagues [19], respectively. In all studies, healthy and periodontitis subjects were diagnosed by a clinician. The datasets included subgingival samples from six healthy individuals and seven individuals diagnosed with periodontitis. One healthy individual (Metagenome_Healthy2) was excluded from the analysis due to its abnormally high level of CBP (0.63 versus a mean of 0.02 for all samples).

### American Indian/Alaskan Native Study Dataset

This dataset included metagenomes generated from 22 subgingival samples from 12 different patients recruited from an American Indian/Alaskan Native population in Southern California [20]. See previous publication for details on sampling methods, disease classification, DNA extraction, and study population [21]. The samples included ten sample pairs from the same patient, one obtained before, and one after standard periodontal treatment. Participants were classified to various degrees of periodontitis based on periodontal pocket depth (PPD), clinical attachment loss (CAL), plaque score, and bleeding on probing (BOP). Individuals with PPD ≤ 4 mm, CAL ≤ 3, and BOP > 10% were classified as having gingivitis; individuals with PPD ≥ 5 mm, CAL ≥ 4, and BOP ≥ 30% were classified as having mild-moderate periodontitis; and individuals with PPD ≥ 7 mm, CAL ≥ 6, and BOP ≥ 30% were classified as having severe periodontitis.

### University of Southern California Study Dataset

This data set includes metagenomes generated from 24 subgingival samples from patients treated at the graduate periodontology clinic at the Herman Ostrow School of Dentistry of the University of Southern California (USC). Information on molecular and clinical methods and study population can be found in a previous study by Califf and colleagues [22]*.* Participants were recruited as part of a study investigating the effectiveness of dilute sodium hypochlorite on periodontitis. Each participant received a comprehensive clinical examination, and was randomly assigned to a control or treatment group [22, 23]. No scaling was performed before or during the treatment. Each patient exhibited at least four separate teeth with a pocket depth of ≥ 6 mm. Pocket depth categories were as follows: class A = periodontal pocket depth up to 6 mm, class b = 6–8 mm, and class c > 8 mm.

### Metagenomic Sequence Processing and Analysis

Metagenome reads were trimmed using the Trimmomatic (v.0.36) [24] default settings (http://www.usadellab.org/cms/?page=trimmomatic). Metagenomes were then subjected to stringent error filtering using PRINSEQ (v.0.20.4) [25] with the following parameters: minimum sequence length of 60 bp, minimum mean quality score of 25, sequences containing any “N’s” were removed, and low-complexity threshold of 50 (using entropy). Human DNA was filtered out using the DeconSeq software (coverage > 90, identity > 90) (v.0.4.3) [26]. After DeconSeq, paired-end files were rewritten to make sure all reads had a mate and separated out singletons using FASTQ Pair, available at https://github.com/linsalrob/EdwardsLab/. BBMerge (v.37.36) [27] was used to merge overlapping pairs of reads using default parameters. Forward reads showed very high quality scores; therefore, those that did not merge were extracted from the bbmerge unmerged output file (https://github.com/pjtorres/xtract_forward) as to not discard useful data.

Metagenomes were analyzed using Kraken [28]. Kraken is able to assign taxonomic labels to short DNA sequences with high sensitivity and speed by utilizing exact alignment of short subsequences of length *k*, called *k*-mers (default size *k* = 31), and a novel classification algorithm. Kraken first uses a reference database and builds a new database by adding phylogenetic information to every *k*-mer in its database. Kraken then classifies reads by breaking each read into overlapping *k*-mers. Each *k*-mer is then mapped to the lowest common ancestor of the genomes containing that *k*-mer in the precomputed database. For our study, we built a custom database containing all complete bacterial reference genomes from the NCBI refseq database (ftp://ftp.ncbi.nlm.nih.gov/genomes/refseq/bacteria/) and CBP.

### Statistical Analysis

Pearson’s product-moment correlation was performed when analyzing CBP relative abundance over pocket depth using the RStudio statistical package (version 1.0.153). Kruskal-Wallis nonparametric tests were used to determine whether the relative abundance of CBP, *P. gingivalis*, or *F. nucleatum* differed between two groups and this was followed by post-hoc Dunn’s multiple comparisons test when comparing three or more groups.

### Genome Mining of Virulence Factors and Biosynthetic Gene Clusters

JGI IMG genome portal analysis pipeline (https://img.jgi.doe.gov/) was used to assess virulence properties of the CBP genome. The antiSMASH tool (the Bacterial version) [29] was applied to search the genome for biosynthetic gene clusters. Default settings were applied.

## Results and Discussion

**Fig. 1:**
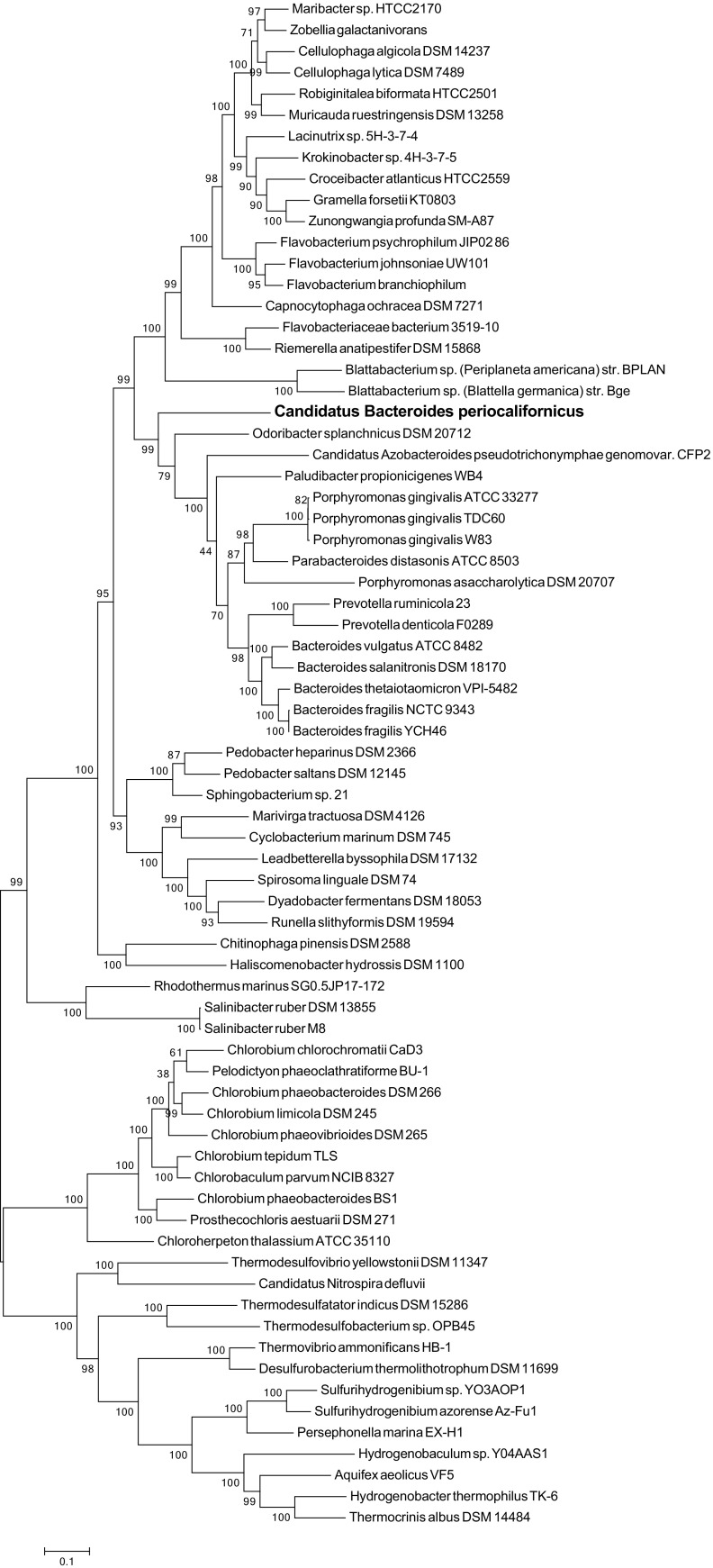
Phylogenetic relatedness of CBP to bacterial lineages within the phylum *Bacteroidetes*. Phylogenetic tree from Maximum Likelihood estimation of 31 marker genes [16] representing 78 different lineages of bacteria within the Bacteroidetes phylum. Five thousand bootstrap iterations were performed. The analysis placed CBP (boldface in figure) deeply within the *Bacteroides*, as sister-group to the genera *Odoribacter*, *Paludibacter, Porphyromonas*, *Parabacteroidetes*, and *Prevotella*

We used the metagenomic taxonomic classification tool Kraken [28] to investigate the relationship of the uncultivated CBP to periodontal disease and to members of key periodontal pathogens in the orange and red complex. CBP had previously been identified in a metagenome from a patient with severe PD in high relative abundance [5]. By performing Maximum Likelihood estimates using multiple marker genes from this genome and 78 additional sequenced genomes, representing a broad diversity of bacteria within the Bacteroidetes phylum; we found that CBP was deeply placed within the *Bacteroides*, as sister-group to the genera *Odoribacter*, *Paludibacter*, *Porphyromonas*, *Bacteroides*, *Parabacteroidetes*, and *Prevotella* (Fig. 1). Its closest neighbor genome was *Alistipes putredinis* with an average nucleotide identity of 68% [5].

**Fig. 2:**
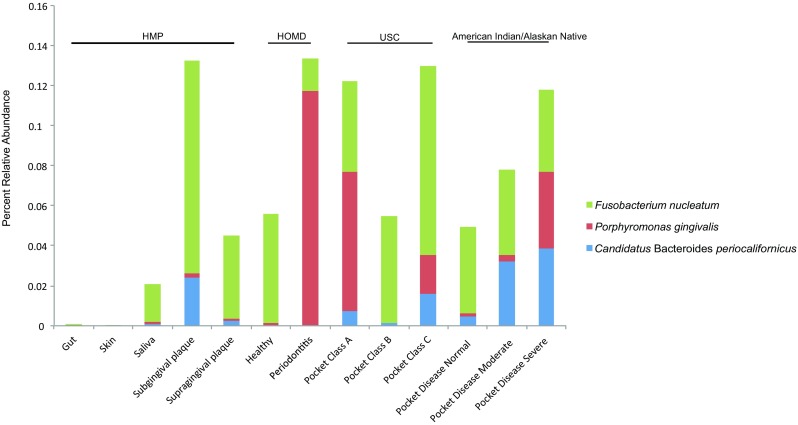
*B*.*periocalifornicus* (CBP) detected in oral sites but not the skin or gut. Stacked bar plot representing the relative abundance (%) of CBP, *P. gingivalis*, and *F. nucleatum* from a combined total of 272 metagenomes generated from different body sites, including gut (*n* = 18), skin (*n* = 14), saliva (*n* = 6), sub- (*n* = 16), and supragingival plaque (*n* = 160) from the HMP datasets; healthy individuals (*n* = 5) and individuals diagnosed with periodontitis (*n* = 7) from HOMD datasets [19]; oral disease class A (*n* = 10), disease class B (*n* = 4), and disease class C (*n* = 10) from USC dataset [22]; oral pocket disease state ranging from normal (*n* = 3), moderate (n = 10), and severe (*n* = 9) from the American Indian/Alaskan Native dataset [21]

**Fig. 3:**
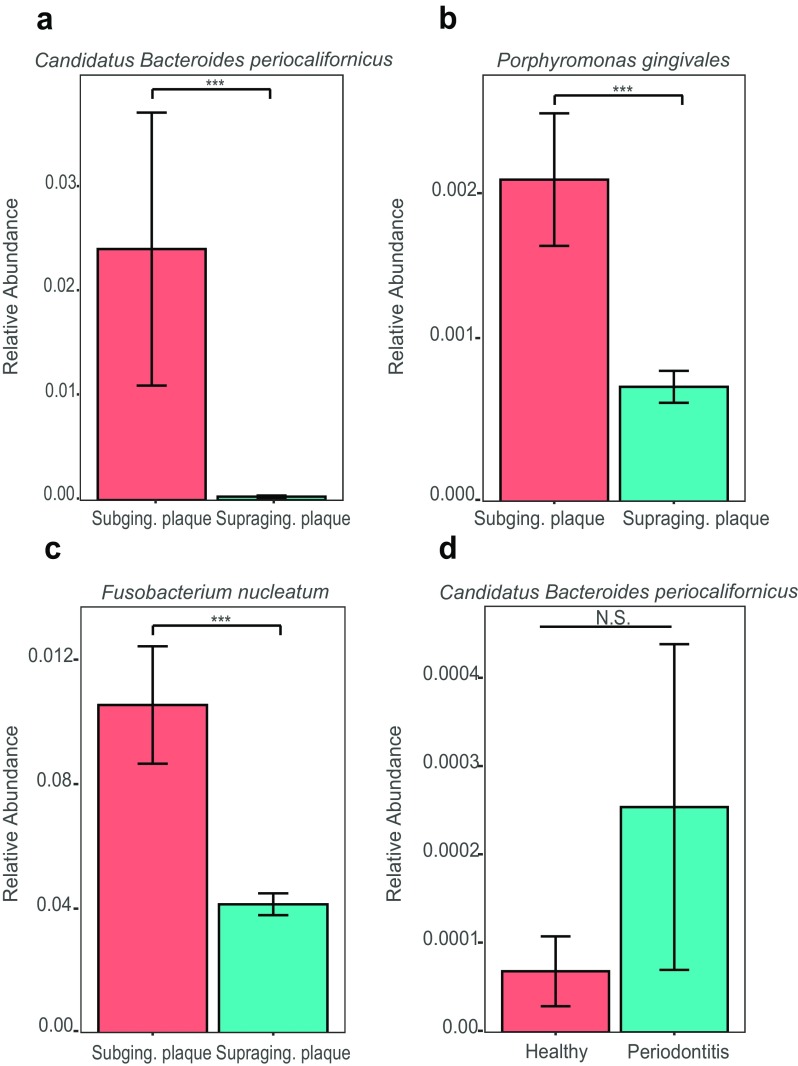
Relative abundance comparisons of *B*. *periocalifornicus* (CBP) in subgingival plaque. Relative proportions of CBP in healthy individuals was compared to individuals diagnosed with periodontal disease (PD), as well as proportions of *P. gingivalis* and *F. nucleatum.* CBP is highly abundant in subgingival as compared to supragingival plaque. Bar plot (mean ± SEM) showing the relative abundance of **a** *B*. *californicus*, **b** *P. gingivalis*, and **c** *F. nucleatum* in sub- (*n* = 16) and supragingival plaque (*n* = 160). Datasets were obtained from the publicly available National Institutes of Health Human Microbiome Project. Dataset for the relative abundance of **d** CBP in subgingival samples from healthy individuals (*n* = 5) and individuals diagnosed with PD (*n* = 7) were obtained from the Human Oral Microbiome Database (HOMD) under the submission number 20130522 (Table S1). (ftp://ftp.homd.org/publication_data/20130522/). Kruskal-Wallis nonparametric test was performed to compare means among the two groups; *** *p* ≤ 0.001

To deepen our understanding of the ecology and biogeographic distribution of CBP, we used the Kraken tool to determine its general abundance in the oral cavity and whether it was specific to the oral cavity or found commonly in other niches of the human microbiome. We analyzed a total of 272 previously published metagenomes from various human body sites representing both healthy and non-healthy adults (Table S1), including: gut (*n* = 18), skin (*n* = 14) saliva (*n* = 6), sub- (*n* = 21) and supragingival plaque (*n* = 160), and adults with periodontal disease (PD) (*n* = 53). By performing multiple comparative nonparametric statistical tests and correlation analyses on the normalized relative abundance values obtained by Kraken, we found that CBP was unique to the oral cavity (Fig. 2), and that the relative abundance of CBP was significantly higher in subgingival plaque compared to supragingival plaque and saliva samples (Fig. S1 and Fig. 3a). These results strongly indicate that CBP is specifically adapted to the subgingival environment, where its relative abundance 0.024 (2.4%) is similar to that of *P. gingivalis* 0.002 (0.2%) and *F. nucleatum* 0.01 (1.0%) (Fig. 3b, c). Comparisons of sequence libraries between healthy subjects and subjects with PD found CBP to be enriched in patients with disease (Fig. 3d).

**Fig. 4:**
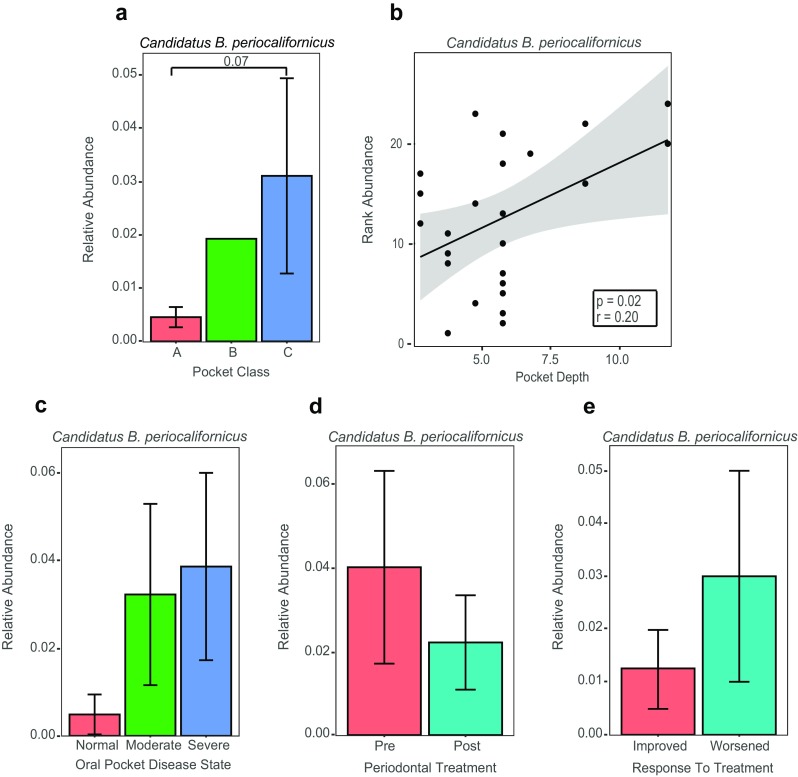
Relative abundance of *Candidatus* *B*. *periocalifornicus* (CBP) in patients with severe periodontal disease (PD). Relative abundance of CBP increases with worsening oral disease class, pocket class, pocket depth and is more abundant in patients with severe oral pocket disease state; decreased posttreatment. Bar plot (mean ± SEM) showing the relative abundance of *Candidatus* Bacteroides *periocalifornicus* from 24 subjects grouped based on **a** Subjects were also grouped based on pocket class A (*n* = 18), pocket class B (*n* = 1) and pocket class C (*n* = 5) (Kruskal-Wallis, *p* value = 0.07). **b** Scatterplot and trend line showing the relationship between pocket depth and ranked CBP relative abundance. Results of Pearson’s correlation (*p* value = 0.02) and correlation coefficient are shown in box inset with gray shaded area indicating the 95% confidence interval for the line of best fit. Bar plot (mean ± SEM) representing the relative abundance of CBP from 22 subjects based on **c** oral pocket disease state ranging from normal (*n* = 3), moderate (*n* = 10), and severe (*n* = 9) (Kruskal-Wallis, *p* value = 0.37). **d** There were 11 subjects in the pre- and 11 in the posttreatment groups [22] (Kruskal-Wallis, *p* value = 0.65). **e** Five subjects improved after standard periodontal treatment and six worsened (Kruskal-Wallis, *p* value = 0.47). Kruskal-Wallis nonparametric tests followed by post hoc Dunn’s multiple comparisons tests were used when comparing three groups. One-way ANOVA on ranked data was used to compare the means between the two groups

**Fig. 5:**
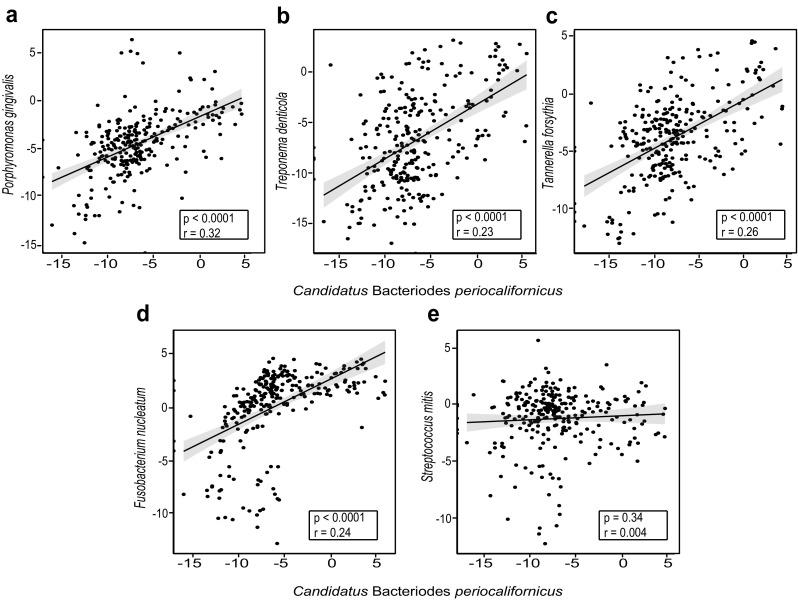
*Candidatus* *B.* *periocalifornicus* (CBP) relative abundance is strongly correlated with all three members of the red complex. Scatterplot and trend line showing the relationship between CBP and **a** *P. gingivalis*, **b** *T. denticola*, **c** *T. forsythia*, **d** *F. nucleatum,* and **e** *S. mitis.* Results of Pearson’s correlation (*p* value and correlation coefficient) are shown in the box inset with gray shaded area indication the 95% confidence interval for the line of best fit (*n* = 272). Relative abundance data was log 2 transformed (normalized)

The association of CBP and the periodontal pocket milieu was further explored by analyzing the presence of CBP in sequence libraries obtained from subjects with different levels of severe PD (detailed information on how PD was diagnosed and how samples were collected can be found in a previous study by Califf and colleagues [22]). The mean relative abundance of CBP was greater in deeper pockets (Kruskal-Wallis, *p* = 0.07; Fig. 4a), and a positive correlation between CBP relative abundance and pocket depth was observed (Pearson’s; *p* = 0.02; Fig. 4b), as well as a trend for more severely disease periodontal pockets and a higher relative abundances of CBP (Kruskal-Wallis, *p* = 0.37; Fig. 4c). The latter correlation was not significant, which is likely due to the high within group variability (a common feature of human microbiome sequence data). In addition, by comparing sequence libraries from PD-patients who were subjected to a 0.25% sodium hypochlorite (diluted bleach) treatment, and whose conditions either improved or worsened after treatment [22], a positive trend was observed, showing a higher abundance of CBP in samples before treatment, and in samples that worsened after treatment (Kruskal-Wallis, *p* = 0.65; Fig. 4d, *p* = 0.47; Fig. 4e). The relative abundance of CBP was also significantly correlated with all members of the red complex (Fig. 5a–c) as well as the orange complex member *F. nucleatum* (Fig. 5d). Furthermore, no correlation was observed between the abundance of CBP and *S. mitis*, a common oral commensal bacterium (Fig. 5e). Intriguingly, our comparative metagenomic read abundance analysis approach of the CBP draft genome not only reveals an orally ubiquitous bacterium, specifically adapted to the subgingival plaque environment, but also a novel candidate member of the pathogenic red complex.

To further elucidate the functional capacity of CBP we employed the JGI IMG genome portal analysis pipeline available at https://img.jgi.doe.gov/, and identified a number of virulence-associated genes. This analysis showed that the CBP genome encode the flagellar assembly proteins CheA, CheB, CheR, CheW, and CheY, which are involved in chemotaxis (i.e., direct movement toward an attractant or away from a repellant), suggesting that CBP is motile and also harbors genes that are key in adhesion to a host and in host invasion [30]. The genome also includes multiple genes involved in beta-lactam resistance, which is in line with numerous studies showing that *Bacteroides* species have the broadest spectrum of resistance to commonly used antimicrobial agents, especially to beta-lactam compounds [31]. In addition, the genome harbors the *rfbA*, *rfbB*, *rfbC*, and *rfBCD* genes, which encode enzymes that are involved in the biosynthesis of dTDP-rhamnose for the assembly of lipopolysaccharide (LPS), suggesting that CBP may have antagonist LPS structures, similar to other *Bacteroidetes*, such as *P. gingivalis* and *Tannerella* [32]*.* Two antioxidant enzymes were also identified (a peroxiredoxin, and a 1-Cys-peroxiredoxin), which are known to control cytokine-induced peroxide levels and are thereby mediating signal transduction in mammalian cells [33]. Furthermore, by performing BLAST analysis of the CBP genome against the well annotated *P. gingivalis* ATCC 33277 genome, we identified the following shared virulence-associated genes: C25 domains encoding gingipains, which are well-known *P. gingivalis* proteases that target outer membranes via the *Bacteroidetes*-specific type 9 secretion system, *ragA* and *ragB* surface antigen genes, and hemolysin encoding genes (Table S2). To further explore the capacity of CBP to produce bioactive small molecules, we applied the antiSMASH software [29]. This analysis predicted that the genome harbors seven putative biosynthetic gene clusters (Table S3) of which one was associated with S-layer glycan biosynthesis, that supports glycosylation of proteins, and gives the cell membrane fluidity, i.e., it is important for gliding motility. Another cluster encode an arylpolyene-like molecule, which corresponds to flexirubin—a pigment associated with all *Bacteroidetes* bacteria, and that is known to protect the cell from oxidative stress [34]. An O-antigen biosynthetic gene cluster, encoding a group of molecules that is known for being important in interactions with other bacterial cells and with human host cells was also identified [35].

Based on all the above findings, we suggest that the CBP genome harbors several genes and pathways similar to the other known oral pathogens belonging to the *Bacteroidetes* phylum, which are involved in PD-associated virulence, including host cell modulation, which strengthens the evidence that this bacterium is a new member of the red complex. A goal is to further elucidate CBP’s role in oral health and disease by attempting various cultivation approaches which, if successful, would allow us to study CBP in the research laboratory and to obtain a complete genome sequence. Also, by applying fluorescent staining techniques, targeting CBP and other oral bacteria, we could learn more about its spatial distribution and physical interactions with other biofilm community members.

## Electronic supplementary material

Table S1 NCBI sequence read archive (SRA) of HMP metagenomic libraries included in this study. The table presents libraries obtained from healthy sampling subjects from the gut, skin, sub- and supragingival plaque. (XLSX 6 kb)

Table S2 BLAST analysis comparing gene homology between *Candidatus* *Bacteroides* *periocalifornicus* and *pathogenic Porphyromonas gingivalis* ATCC 33277. (XLSX 174 kb)

Table S3 Results from antiSMASH analysis showing *Candidatus* periocalifornicus genomic potential to produce bioactive small molecules. (XLSX 40 kb)
